# Segmentation-based lightweight multi-class classification model for crop disease detection, classification, and severity assessment using DCNN

**DOI:** 10.1371/journal.pone.0322705

**Published:** 2025-05-14

**Authors:** Chatla Subbarayudu, Mohan Kubendiran

**Affiliations:** School of Computer Science and Engineering, Vellore Institute of Technology, Vellore, India; Shandong Agricultural University, CHINA

## Abstract

Leaf diseases in Zea mays crops have a significant impact on both the calibre and volume of maize yield, eventually impacting the market. Prior detection of the intensity of an infection would enable the efficient allocation of treatment resources and prevent the infection from spreading across the entire area. In this study, deep saliency map segmentation-based CNN is utilized for the detection, multi-class classification, and severity assessment of maize crop leaf diseases has been proposed. The proposed model involves seven different maize crop diseases such as Northern Leaf Blight *Exserohilum turcicum*, Eye Spot *Oculimacula yallundae*, Common Rust *Puccinia sorghi*, Goss’s Bacterial Wilt *Clavibacter michiganensis subsp. nebraskensis*, Downy Mildew *Pseudoperonospora*, Phaeosphaeria leaf spot *Phaeosphaeria maydis*, Gray Leaf Spot *Cercospora zeae-maydis*, and Healthy are selected from publicly available datasets obtained from PlantVillage. After the disease-affected regions are identified, the features are extracted by using the EffiecientNet-B7. To classify the maize infection, a hybrid harris hawks’ optimization (HHHO) is utilized for feature selection. Finally, from the optimized features obtained, classification and severity assessment are carried out with the help of Fuzzy SVM. Experimental analysis has been carried out to demonstrate the effectiveness of the proposed approach in detecting maize crop leaf diseases and assessing their severity. The proposed strategy was able to obtain an accuracy rate of around 99.47% on average. The work contributes to advancing automated disease diagnosis in agriculture, thereby supporting efforts for sustainable crop yield improvement and food security.

## 1. Introduction

With the increase in the world population, there is a corresponding rise in the need for food. Unfortunately, the inability to produce enough food to meet this demand is a significant global issue. Food shortages are mostly caused by agricultural losses resulting from illnesses, severe weather conditions, and a lack of resources. These factors all add to the problem. Projections predict that the global population will increase by roughly 2 billion, and by 2050 there will be an increase from 7.7 billion to 9.7 billion [1]. To fulfill the needs of a growing population, we must preserve long-term food security. Maize, sometimes known as “corn,” is a crop that is quickly gaining popularity and can thrive in many climatic situations due to its outstanding adaptability. It holds significant importance both as a food source and in industrial applications worldwide. Known as the “queen of cereals,” maize boasts the highest yield among cereal crops. Maize is extensively cultivated globally and serves as a vital food crop as well as an industrial raw material. After wheat and rice, Maize is a highly significant cereal crop on a global scale [2]. Despite its excellent output, maize is extremely susceptible to different diseases during the growing season. Thus far, researchers have detected approximately 112 diseases in maize crops from various locations across the globe [3]. Maize disease is caused by various viruses, viroid, fungi, bacteria, and other pathogens, which are often referred to as physiological lesions. These diseases can significantly impact maize crop yields, potentially causing moderate to severe damage. Although the maize plant has a high potential for producing a large yield, it is susceptible to many illnesses that can result in a yearly decline of 6%–10% [4]. Currently, several diseases such as grey leaf spot, northern leaf blight, Cercospora leaf spot, common rust leaf, phaeosphaeria leaf spot, maize eyespot, gross bacterial wilt, sheath blight and banded leaf, brown spot, southern leaf blight, curvularia leaf spot, dwarf mosaic, rust, and round spot are more prevalent. Precise and timely identification of maize diseases is essential for improving crop yields, as it enables effective monitoring and treatment of crops. Since farmers lack specialized knowledge, accurate diagnosis of maize leaf diseases is essential to ensure productivity. Traditionally, identifying these diseases involves manual leaf inspection, relying on the expertise of agricultural specialists. Incorrect diagnoses often lead to ineffective pesticide use, which pollutes the environment and increases toxicity in maize. Therefore, rapid and accurate methods are necessary for monitoring and treating maize infections.

Maize is susceptible to various foliar diseases that significantly impact crop yield and quality. A brief overview of the pathogens responsible for these diseases enhances the biological context and aids future research. Northern Leaf Blight is caused by *Exserohilum turcicum*, a fungal pathogen known for its characteristic elongated lesions on maize leaves. Common Rust, triggered by *Puccinia sorghi*, presents as reddish-brown pustules that spread under favourable conditions. Eye Spot, attributed to *Aureobasidium zeae*, results in circular, water-soaked lesions that can compromise photosynthesis. Downy Mildew, a group of diseases caused by various species of *Peronosclerospora*, leads to chlorotic streaking and systemic infection in maize plants. Goss’s Bacterial Wilt, induced by *Clavibacter michiganensis subsp. nebraskensis,* manifests as dark, water-soaked streaks accompanied by bacterial exudates. Gray Leaf Spot, caused by *Cercospora zeae-maydis*, is characterized by rectangular, necrotic lesions that reduce photosynthetic efficiency. Lastly, Phaeosphaeria Leaf Spot, associated with *Phaeosphaeria maydis*, produces small, dark lesions that can coalesce under severe infections, further impacting plant health.

**Fig 1:**
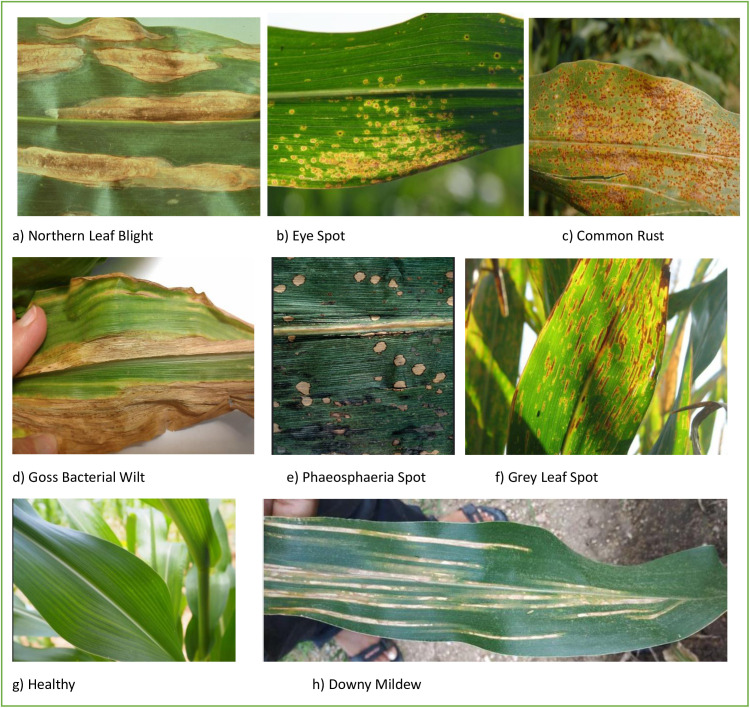
Images of maize leaf illness sample.

Detecting disease symptoms early in crops is a significant advancement in tackling both economic and environmental issues [5]. Identifying diseases in maize crops has become increasingly challenging, necessitating advanced expertise to recognize symptoms that are not yet fully developed. Therefore, there is a need for automated and intelligent systems capable of diagnosing crop diseases accurately and efficiently. Leveraging advances in computer vision technology, researchers have developed numerous machine learning algorithms to diagnose plant diseases [6–8]. However, the study’s choice of classification technique limits the capabilities of the model [6,7]. In recent years, advancements in artificial intelligence (AI) have led to substantial progress across various fields, including agriculture. Recently, the introduction of deep learning-based disease diagnosis has revolutionized plant disease identification, offering high accuracy and autonomy, and has been rapidly applied to maize and other crops. These methods provide robust solutions to challenges faced by traditional machine learning approaches, effectively addressing maize diseases with the aid of growing data and advanced hardware [9]. Deep learning (DL) systems, which are a subset of machine learning techniques, have gained popularity due to their extraordinary skills in recognizing and classifying patterns. Convolutional neural networks (CNNs), DL techniques, including recurrent neural networks (RNNs) [10], and deep belief networks [11], have been extensively used in various fields, including agriculture, for tasks like predicting yield amounts, identifying plants, and diagnosing crop diseases [12]. Deep learning has proven to be important in numerous fields, including healthcare, agriculture, autonomous vehicles, defense, and many other industries [13–15]. Fig 1 presents sample maize leaf disease images from various disease categories.

Nevertheless, only a restricted group of researchers concentrated on evaluating the intensity of plant diseases by combining image-based analysis with machine learning models. The researchers employed support vector machines (SVM) to detect and categorize Bacterial Sheath Blight, Leaf Blight, and Blast in rice by analyzing visible pictures [16]. The study utilized a combination of advanced statistical methods, including Gaussian process regression, partial least support vector regression, and squares regression, to predict the severity of wheat leaf rust from hyperspectral readings [17]. Researchers developed a system that utilizes deep learning to examine images of wheat leaves captured by mobile phones, and then the system can estimate the level of yellow rust severity on the crops [18]. Scientists investigated a different approach combining convolutional neural networks (CNNs) and support vector machines (SVMs) to identify and categorize brain tumors from MRI scans [19]. Researchers proposed a new disease detection system that merges CNNs and LSTM networks to classify three common rice leaf detections namely blast, bacterial blight, and leaf smut [20].

Most models on segmentation and classification suffers from problems such as noise and irrelevant features. The models lack sufficient quantity of datasets which is a tedious and time-consuming process [21–23]. Hence in order to reduce the noise, irrelevant features, and computational complexity and to improves the generalization, interpretability and versatility deep saliency-based region segmentation has been utilized.

### 1.1 Motivation

Maize, a staple crop worldwide, is crucial for food security and economic stability in many places. However, its productivity is significantly threatened by various leaf diseases, which can lead to substantial yield losses and economic hardship for farmers. Effective management of maize leaf diseases is imperative to ensure sustainable agricultural practices and food supply. Although agricultural science has made significant progress, the early and precise identification of these illnesses continues to be difficult because of the intricate symptoms and the fast dissemination of infections. Conventional illness diagnosis procedures, which often rely on visual examinations conducted by specialists, are time-consuming and susceptible to human mistakes. The advancement of innovative and efficient disease detection techniques is therefore of paramount importance. Utilizing modern technologies such as image processing, machine learning, and remote sensing can revolutionize the way we identify and manage maize leaf diseases. These technologies offer the potential for early detection, which is crucial for timely intervention and minimizing crop damage. In this context, Deep Saliency based segmentation has been adopted for region segmentation, providing a powerful tool for accurately identifying diseased areas on maize leaves.

This research intends to bridge the gap between traditional agricultural practices and modern technological advancements by developing robust methods for the classification and detection of maize leaf diseases. By leveraging advanced computational techniques, this study seeks to provide farmers with reliable tools to protect their crops, improve yields, and contribute to the overall stability of the global food supply.

### 1.2 Major contributions

The proposed segmentation-based lightweight crop disease detection and multi-class classification model involves the following contributions which are summarized as follows:

- The proposed technique involves the use of image pre-processing techniques such as image augmentation, and `CLAHE to improve the quality of the image. Further, Deep Saliency based segmentation has been adopted for region segmentation to alleviate the issues of overfitting and noise removal.
- After the disease-affected regions are identified the features are extracted by using the EfficientNet-B7 a pre-trained deep learning architecture. Additionally, Bayesian optimization is utilized for hyper-parameter optimization. After the features are extracted, a hybrid harris hawks’ optimization (HHHO) is utilized for feature selection. The simulated annealing (SA) technique is utilized for controlling the convergence rate to select the predominant features.
- Finally, from the optimized features obtained, classification is performed with the help of Fuzzy SVM.
- Performance analysis has been carried out by the use of maize leaf disease datasets obtained from commercially available public datasets utilizing metrics such as accuracy, precision, recall, and severity assessment factors. To ascertain the efficiency of the proposed work, a comparative analysis along with the chosen benchmarks are analysed and reviewed. Further ablation study has been provided in order to ascertain the effectiveness of the proposed approach.

### 1.3 Organization of the work

The proposed work involves the abstract followed by the introduction, motivation, and major contributions. Further to support the study several literary works pertaining to crop disease detection and classification are analysed and reviewed. Further, a segmentation-based lightweight classification model has been proposed followed by the experimental analysis, and concluded with accuracy and severity assessments. Table 1 provides the chronicle of abbreviations utilized in our proposed work.

**Table 1:** Chronicle of abbreviations.

Acronym	Abbreviation
DCNN, CNN	Deep Convolutional Neural Network, Convolutional Neural Network
CLAHE, DenseNet	Contrast Limited Adaptive Histogram Equalization, Dense Neural Network
VGG, SVM, ResNet	Visual Geometry Group, Support Vector Machine, Residual Network
LCNN, CBAM	Lightweight Convolutional Neural Network, Convolutional Black Attention Module
HHHOSA, ADAM	Hybrid Harris Hawks Optimization with Simulated Annealing, Adaptive Moment Estimation
CR, NCLB, GLS	Common Rust, Northern Corn Leaf Blight, and Gray Leaf Spot
GAN, RCNN	Generative Adversarial Networks, Region-based Convolutional Neural Network
MGWO, MaxViT, SA	Modified Grey Wold Optimization, Multi-axis Vision Transformer, Simulated Annealing
ES, ASR, GSR, ALB	Eye Spot, Anthracnose Stalk Rot, Gibberella Stalk Rot, Anthracnose Leaf Blight,
SR, NCLS	Southern Rust, Northern Corn Leaf Spot
ClGan, CAD	Crop Leaf GAN, Computer-aided system
SKPS	Select Kernel–Point–Swish

## 2. Related works

Several works have been contributed by various researchers pertaining to crop disease detection and classification. Most of these schemes utilize CNN models in combination with different machine learning, and optimization techniques to achieve high accuracy and to reduce the computational cost involved in classification. This section provides a list of related works about different crop diseases along with comparative analysis in terms of problems addressed, solutions, benefits, and limitations.

### 2.1 Classification of various works on crop disease detection and classification

Poornima Singh et al. [24] introduced an efficient lightweight Convolutional Neural Networks model for crop identifying crop illness. Their proposed system involves the use of an automatic VGG-ICNN plant disease identification model, which has been trained on five distinct public Plant Village datasets encompassing various crop varieties, such as apples, maize, and rice. Their model reached an accuracy of 99.16%. Nonetheless, to be suitable for real-time deployment, the system requires a reduction in the model size. An end-to-end deep learning model for the categorization of maize leaf diseases was proposed by Hassan Amin et al. [25]. Their technique handles the issue of promptly identifying plant illnesses, a task that is arduous and time-consuming when distinguishing between healthy and diseased plants. The proposed system involves the use of CNNs, EfficientNetB0, and DenseNet121 architectures with data augmentation techniques. The model attained an accuracy of 98.56%, whereas the Resnet152 model reached 98.37% and the inceptionV3 model obtained 96.26%. However, more enhancements to the recommended model may be made by studying further augmentation approaches and experimenting with alternative combinations of CNNs as feature extractors. Ashraful Haque et al. [26] proposed deep learning-based models for the recognition of maize crop diseases. Their research utilized 3852 maize crop images sourced from the Plant Village data repository. The proposed method addresses three types of diseases: Northern Corn Leaf Blight (NCLB), Common Rust (CR), and Gray Leaf Spot (GLS). Their model attained an accuracy of 99.10%. However, the proposed method still faces challenges when trained and tested on images of diseased crops taken in natural background conditions, and it also struggles to identify multiple diseases in other significant crops. Shaodong Cui et al. [27] have come up with maize leaf disease of a lightweight autoencoder classification model using a Convolutional Black Attention Module (CBAM) their proposed system involved image reconstruction techniques to enhance feature interpretability, and the discrete wavelet transform was used to perform initial dimensional reduction on the images. Their proposed model achieved an accuracy of 99.44%. Nevertheless, the proposed model needs to be used for classifying other crop leaf diseases.

Wilbur et al. [28] their proposed a model for maize crop analysis obtained through drone flights, which was developed and validated based on multispectral image analysis. Their proposed system involves pattern recognition methods utilizing distance maps, with a particular emphasis on the application of Euclidean distance. Feature extraction was performed using chain code and circular pattern map techniques, and classification was conducted using a support vector machine (SVM). Their system achieved a success rate of 88.47%; however, the accuracy still needs to be improved. Additionally, there is a need to enhance the application of sensors that capture depth information from aerial images, in combination with the use of semantic segmentation techniques and artificial intelligence. Vivek Sharma et al. [29] introduced an innovative approach for identifying maize leaf diseases by employing ClGan (Crop Leaf GAN) alongside deep CNN models. Their research addresses the challenge of the larger parameter size of deep learning models, which is especially difficult for agricultural due to the limited resources available. The model they proposed achieved an accuracy of 99.97% in training and 99.04% in testing. The advantages of the proposed system include an improved loss function and the introduction of a dynamic correction factor. The comparison included five other advanced GAN models: W-GAN, DC-GAN, WGAN-GP, InfoGAN, and LeafGAN. Despite this, ClGan-Net requires further testing on a wider range of plant leaf datasets and with unmanned aerial vehicles to detect illness images in real-time environments and cyber-physical systems. Momina Masood et al. [30] the model they proposed a deep learning method to efficiently recognize infections in maize plant leaves. Their system was designed to monitor and identify infections during the planting season, tackling challenges in real-world conditions, including significant distortions, such as noise, background clutter, and blurring of the leaf region. Their system used a Faster R-CNN approach that utilizes the ResNet-50 model and obtained an accuracy of 97.89%. Nevertheless, the proposed system needs to improve its performance accuracy when evaluated on other challenging datasets.

Helong Yu et al. [31] presented an image segmentation method using a multi-stage Cauchy-enabled Grey Wolf algorithm for detecting leaf spot disease in maize. Their proposed system involves improving the MGWO’s ability to optimize while ensuring a specific rate of convergence. The disadvantage of the proposed system is the time complexity, which arises without reducing the effectiveness of the algorithm. However, the proposed methodology needs to be improved to address optimization challenges in various domains, which require further validation. Weihui Zeng et al. [32] developed a convolutional neural network model called SKPSNet-50 to identify illnesses in maize leaves. Their approach utilized the integrated focal loss function to direct the parameter optimization of the model, addressing the issue of data imbalance. They used 1,452 images of maize crops obtained from the PlantVillage data repository for their research. The proposed method addresses five types of diseases: Downy mildew, Leaf blight, Rust, Corn borer eating, Locust eating, and Healthy. Nonetheless, the suggested system needs to focus on the efficient use of network model parameters, develop a lightweight network for smartphones, and create an app. Rajeev Kumar Singh et al. [33] proposed using deep transfer modelling to classify diseases in maize plant leaves. Their model employs the AlexNet architecture, trained on a dataset of 1,363 maize plant images from PlantVillage. By using DNN and the AlexNet model, maize diseases are classified to identify illnesses in maize leaves. The model targets two categories of diseases: those resulting from leaf-spot infections, such as Cercospora and Gray Leaf Spot, and diseases caused by common rust. The advantages of the proposed system include the ability of the CNN to automatically extract features by processing the raw images directly. Their proposed model has obtained an accuracy of 99.19%; however, the proposed system still suffers from a low dataset size.

In their study, Ishak Pacal et al. [34] proposed improving crop yield and sustainability by detecting diseases in maize leaves. They leveraged a massive dataset using CNN-based and advanced vision transformer models. In this study, a computer-aided system (CAD) is crucial in agriculture, facilitating timely and efficient disease identification. Their proposed system involves a multi-axis vision transformer (MaxViT) architecture, which was evaluated on separate test datasets comprising 3839 images (PlantVillage + PlantDoc + CD&S). The accuracy of the proposed model is an impressive 99.24%. Gayathri Devi et al. [35] proposed using an AlexNet-Inception network model to analyze and classify diseases in maize crops. Their proposed system involved different optimization methods, such as RMSprop (Root Mean Squared Propagation), Stochastic Gradient Descent with Momentum (SGDM), and Adaptive Moment Estimation (ADAM), which were used to train the network. Their model classified the dataset is divided into four distinct classes: blight, common rust, grey leaf spot, and healthy. These classes were trained using multiple hyperparameters, such as learning rates and mini-batch sizes. The advantages of the proposed methodology include the network becoming progressively wider, not deeper, which reduces the computational cost and avoids the vanishing gradient problem. The proposed method achieved an accuracy of 98.91%. Nagaraju et al. [36] developed a method for detecting diseases in maize crops using the NPNet-19 convolutional neural network. Their proposed system was designed to detect disease, process images, and perform disease recognition and classification. The technique utilized an expanded dataset consisting of 15,960 images from PlantVillage and Kaggle, used to classify six disease classes: Southern Rust (SR), Anthracnose Stalk Rot (ASR), Gibberella Stalk Rot (GSR), Eye Spot (ES), Northern Corn Leaf Spot (NCLS), Anthracnose Leaf Blight (ALB), and Healthy. Their proposed system was compared with pre-trained models, including ShallowNet-8, DenseNet-121, Inception V2, and CNN-SVM. Despite achieving an accuracy of 99.19%, the proposed model still has issues with identifying and categorizing illnesses in real-time.

Ramar Ahila Priyadharshini et al. [37] proposed a method that uses deep convolutional neural networks to classify maize leaf diseases. Their system aimed to identify diseases rapidly, addressing the challenge posed by inadequate infrastructure in many regions. The proposed methodology involves using a modified LeNet architecture and an expanded dataset of 3,852 images from PlantVillage to classify four different classes: Gray Leaf Spot, Northern Leaf Blight, Common Rust, and Healthy. The proposed system model obtained an accuracy of 97.89%; nevertheless, there is still potential for further enhancement in accuracy. Poonam Dhiman et al. [38] proposed a novel deep-learning model for detecting the severity level of diseases in citrus fruits. Their system utilized a detailed analysis and graph-based segmentation in its approach, which were then applied for selective search. The model was trained to identify specific areas affected by the disease and classify them into four categories: high, medium, low, and healthy. Additionally, the model implemented multi-classification for each severity level using transfer learning with VGGNet. Their proposed method achieved prediction accuracies of 96% for healthy, 99% for low level, 98% for high level, and 97% for medium levels of disease. Wu et.al. [39] addressed the problem of tiny object loss and distinguishability with the increase in the depth of the neural network. Their methodology involves the use of U-Net in U-Net framework to detect short and small objects in infrared images. Their proposed technique provides the feature training from both the global and local information. Their methodology further integrates resolution maintenance deep supervision (RM-DS) and the interactive cross attention (IC-A) module along with the residual U-block module. Their system has been experimented by using SIRST and synthetic datasets. Their model achieved loss mitigation and can be able to distinguish without relying on the backbone architecture. However, the accuracy of the model needs to be increased. Their proposed model utilized video sequence as input. In case of static low-resolution images, the models needed to be evaluated. Wu et.al. [40] proposed a cross-channel reconstruction module based deep learning framework for multimodal remote sensing data. Their work has been aimed to improve the performance of classification approach utilized. Their method has been tested by using datasets such as hyperspectral and LiDAR dataset (Houston2013) and the SAR dataset (Berlin). Their method generates more realistic and smoother classification maps with less noisy pixels. However still there exists a need to enhance its accuracy. Li et.al. [41] has come up with a Cascaded transformers for fusion-aware computational hyperspectral imaging technique. Their methodology addressed the problem of ill-poised blind reconstruction process during imaging. It involves cascaded attention blocks with decoupling loss function, thereby ensuring spatial consistency and spectral fidelity. However still there is a need to address parallax elimination and image registration. Table 2 provides a comparative analysis of the relevant research.

**Table 2:** Comparative analysis of various works pertaining to crop disease detection and classification.

Ref	Problem Addressed	Solution	Dataset	Benefits	Limitations
[24]	Light weight CNN model for crop disease identification	VGG-ICNN	Public PlantVillage	Obtained an accuracy of 99.16%	The technique needs to reduce the model size to make it suitable for real-time deployment.
[25]	Rapid identification of plant diseases	(CNN), EfficientNetB0, and DenseNet121	PlantVillage dataset hosted on Kaggle	Attained an accuracy of (98.56%) outperforming Resnet152 (98.37%) and InceptionV3 (96.26%)	The model needs to be improved by exploring more augmentation techniques and trying different combinations of CNNs as feature extractors.
[26]	Deep learning-based models for the recognition of maize crop diseases	CNN(DenseNet121)	PlantVillage	Achieved an accuracy of 99.10%	The method still encounters difficulties when applied to diseased images taken under standard background conditions and when recognizing various diseases in other crops.
[27]	Maize leaf disease classification	Lightweight autoencoder classification model using Convolutional Black Attention Module	PlantVillage	Achieved an accuracy of 99.44%	Changing the model’s parameters affects both training accuracy and time.
[28]	Counting maize plants and evaluating crops using multispectral image analysis	Pattern recognition techniques such as feature extraction (SVM) and post-processing	Real-time dataset	Achieved a success rate of 88.47%	Accuracy still requires improvement.
[29]	Maize leaf disease identification	ClGan (Crop Leaf GAN) DC-GAN, W-GAN, *W Gan*_*GP*_, InfoGan, and LeafGan	PlantVillage	Achieved an accuracy of 99.97% in training and 99.04% in testing	Excessive training (epochs) lowers performance. Accuracy first increases, but then plateaus and decreases at 30 epochs, indicating overfitting.
[30]	Background noise, clutter, and blurry leaf regions significantly complicate maize leaf recognition	Faster-RCNN and ResNet-50	Agronomy Center for Research and Education at Purdue University	ResNet-50 model and achieved an accuracy of 97.89%	The model still struggles to increase its accuracy.
[31]	Image segmentation of leaf spot disease on maize	Multi-stage Cauchy-enabled Grey Wolf algorithm	wheat yellow rust (WYR)	ML models successfully predicted wheat rust severity with decent accuracy. This paves the way for a fast method to estimate yellow rust in wheat fields	The limitation of the proposed system is its time complexity, which affects the effectiveness of the algorithm’s performance
[32]	An identification method for maize leaf diseases	convolutional neural network model SKPSNet-50	PlantVillage	To address data imbalance, their system employed a combined focal loss function, guiding model parameter adjustments.	The model should prioritize the efficient use of network model parameters, create a lightweight network for smartphones, and develop an accompanying app.
[33]	Maize plant leaf disease of classification	Deep transfer modeling such as the AlexNet model	PlantVillage	Obtained an accuracy of 99.19%	The proposed system still suffers from a low dataset size.
[34]	Maize leaf disease identification	CNN-based and advanced vision transformer models	PlantVillage	Accuracy reaches an impressive 99.24%	The study combines data from PlantVillage, PlantDoc, and CD&S to create the largest collection of images for identifying maize leaf diseases.
[35]	The study focuses on the classification and feature analysis of agricultural diseases affecting maize	AlexNet-Inception network model	Kaggle wheat dataset	Achieved an accuracy of 98.91%	Despite good results, accuracy gains are likely with variations of Inception modules, attention mechanisms, or integrating shallow machine learning.
[36]	Maize crop disease detection and classification	NPNet-19 convolutional neural network	PlantVillage	Accuracy of 99.19%	The model still faces challenges in better detecting and classifying real-time diseases.
[37]	Maize leaf disease classification	Deep convolutional neural networks (modified LeNet)	PlantVillage	Accuracy of 97.89%	The model still has room for improvement in accuracy
[38]	Detecting the severity level of diseases in citrus fruits	DNN model VGGNet	PlantVillage	Achieved prediction accuracies of 96% for healthy, 99% for low level, 98% for high level, and 97% for medium levels of disease.	The model still struggles to increase its accuracy.
[39]	Loss and Feature Distinguishability	UIU-Net	SIRST, Synthetic	UIUNet also produces powerful generalization performance for video sequence infrared small object datasets	Network need to be optimized for enhanced accuracy and efficiency in detecting objects within complex video scenarios
[40]	Classification performance	CCR-Net	Multi-Modal Datasets, Houston 2013, Berlin Datasets	Achieved an Accuracy of 85.93%	Diverse data modalities has to be investigated further
[41]	Weak Generalization, Strong Coupling and Inadequate data	CasFormer	CAVE, KAIST, and ICVL datasets	Achieved an accuracy of 98.4%	Their approach lacks effective parallax elimination and robust image registration, limiting its performance in unregistered image scenarios. Further optimization of fusion-based imaging architectures and exploration of new fusion strategies are required.

## 3. Methodology

**Fig 2:**
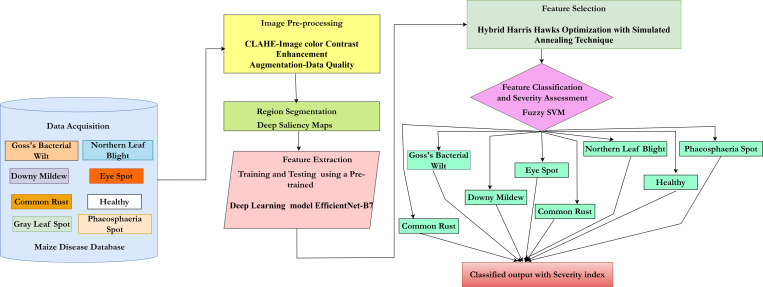
Proposed architecture for maize leaf disease classification.

The work being proposed utilizes transfer learning and deep learning methods for the identification and the multi-class segregation of maize leaf diseases (Fig 2). The methodology involves six different stages viz., image acquisition, image pre-processing, image segmentation, image feature extraction, image feature selection, and Multi-Class Classification. The proposed methodology involves the maize leaf disease images obtained from commercially available public datasets. Firstly, CLAHE [42] is utilized to enrich the color contrast of the image. Further, the quality and the imbalanced nature of distinct classes can be balanced by the use of image augmentation [43]. Deep Saliency Maps [44] are used for segmenting the disease-affected regions from the images. The regions that are segmented are then fed into a pre-trained deep learning model called EfficientNet-B7 [45] for feature extraction. After the feature gets extracted a hybrid harris hawks optimization algorithm [46] is utilized for feature selection. During the training of the network, the hyper-parameters are optimized with the help of the simulated annealing technique [47]. Finally, the images are classified into various classes that perform efficient detection and classification using SVM [48]. In the final step, along with multi-class classification severity of the disease is assessed.

### 3.1 Image acquisition

Maize crop images were obtained from publicly available datasets provided by the PlantVillage repositories, as well as data extracted from published research articles [49–52]. The dataset obtained comprises of seven different classes such as Phaeosphaeria leaf spot, common rust, downy mildew, goss’s bacterial wilt, northern leaf blight, gray leaf spot, eye spot, and healthy image consisting of about 16118 images of maize leaf disease.

Northern Leaf Blight, Common Rust, and Grey Leaf Spot class-type images were gathered from a dataset called PlantDoc. The PlantDoc dataset was created by downloading images from Google and Ecosia. The dataset contains 2,598 images with 27 classes spanning across 13 species. Eye Spot class-type images were collected from a dataset called CLESt Disease, which was gathered from a district called Patiala in the Indian state of Punjab. The dataset contains 6,000 images. Goss’s Bacterial Wilt class-type images were collected from a dataset called Maize Disease Identification. The images in this dataset were captured at the Innovation Farm of the Xiamen Institute of Subtropical Botany, Fujian Province, China. The dataset contains 466 maize crop images with a resolution of 224 × 224 pixels. The Phaeosphaeria leaf spot dataset was collected from maize fields and consists of 2,355 images of maize leaves taken from various locations in South Africa between January 2009 and December 2020. The images were captured using a Nikon D90 SLR camera as well as a variety of smartphones. The dataset consists of images obtained from fields based on the availability of the maize crop over the time of two months, i.e., July and August 2019. Images were captured using the Redmi Note 4 smartphone with a 13MP camera and an aperture of f/2.0. Resizing and cropping of images from 2340 × 4160 native camera resolution to 512 × 512 pixels. The dataset underwent the process of data pre-processing. The details of the datasets are provided in Table 3.

**Table 3:** Sample of dataset.

Dataset	Samples
Northern Leaf Blight	3940
Grey leaf Spot	2052
Goss’s Bacterial Wilt	120
Downy Mildew	350
Common rust	4768
Eye Spot	121
Phaeosphaeria leaf spot	119
Healthy	4648
Total	16118

### 3.2 Image pre-processing

The acquired dataset is thereafter subjected to preprocessing before its use for training and testing purposes. The dataset undergoes preprocessing to optimize color contrast and increase picture augmentation.

#### 3.2.1 Enhancing the color-contrastness.

The color contrastness of the dataset is performed by using the technique called CLAHE. The primary goal of color contrast enhancement is to increase the quality of an image. The photos are separated into little portions known as files, which are used to calculate histogram equations. The essential feature of the technique is that it preserves the characteristic feature of an image by stretching its pixel intensities. This method essentially protects the image pixel being washed out thereby alleviating over-saturation and feature loss. The major significance of CLAHE is that it performs better when it has high depth or low contrast. CLAHE essentially relies on ameliorating the contrastness on the local convergence than at the global convergence. File size and clip limit are the two major hyperparameters of CLAHE. The selection of the hyperparameter determines the image quality. Therefore, a clip limit (5) and a file size 10*10 are selected for histogram equalization.

### 3.3 Image enhancement using augmentation

To balance the number of classes uniformly, image augmentation is performed when the number of input parameters is high to obtain the optimal performance and be enhanced. Image augmentation is highly suited for broad and varied datasets. The proposed methodology involves various image augmentation techniques such as moving, rotating, flipping, and cropping. It increases the performance of the underlying deep learning classification model thereby alleviating the overfitting of data. The proposed work involves 90^0^ rotations 0.2 of width_shift_range, height shift, and horizontal flipping is performed. Table 4 provides the total number of data utilized after augmentation. Hence total of 14828 images are enhanced which can be utilized for maize disease detection. Table 4 provides the details of dataset distribution.

**Table 4:** Dataset utilized.

Classes	Before Augmentation	After Augmentation
Northern Leaf blight	3940	3600
Common Rust	4768	3668
Eye Spot	121	111
Downy Mildew	350	317
Goss’s Bacterial Wilt	120	90
Gray leaf Spot	2052	1942
Phaeosphaeria leaf spot	119	100
Healthy	4648	4400
Total	16118	14228

### 3.4 Image segmentation using deep saliency in crop disease detection

**Fig 3:**
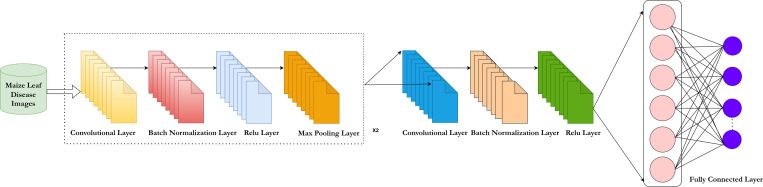
14-Layer convolutional neural network.


Image segmentation is achieved using deep saliency maps. This process utilizes a 14-layered Convolutional Neural Network (CNN) incorporating techniques such as thresholding, convolution, merging, closing, and filling. The CNN architecture includes three batch normalization layers, two activation layers, one average pooling layer, one fully connected layer, and a SoftMax classifier. The network uses a stride of 2x2 and a filter size of 3x3. Training involves a learning rate of 0.05, 100 epochs, a batch size of 32, a momentum of 0.6, a dropout rate of 0.5, and the HHHO optimizer. Fig 3 illustrates the 14-layered CNN architecture. Equation (1) shows the output of the region segmentation using deep saliency maps, where the original infected saliency map is represented. Fig 3 provides the architecture of CNN utilized for deep saliency map-based segmentation.

$${\hat{C}}_{map}=\{C_{map+\sum i=1\gamma_{1}(\overset{―}{\rho })}\}$$ (1)

This map indicates the original image’s saliency and an enhanced version is denoted as ${\hat{C}}_{map}$. $C_{map}$ can be used to identify the infected region in the saliency map, while $\overset{―}{\rho }$ is the image-enhanced version.

### 3.5 Image extraction using EfficientNet-B7

**Fig 4:**
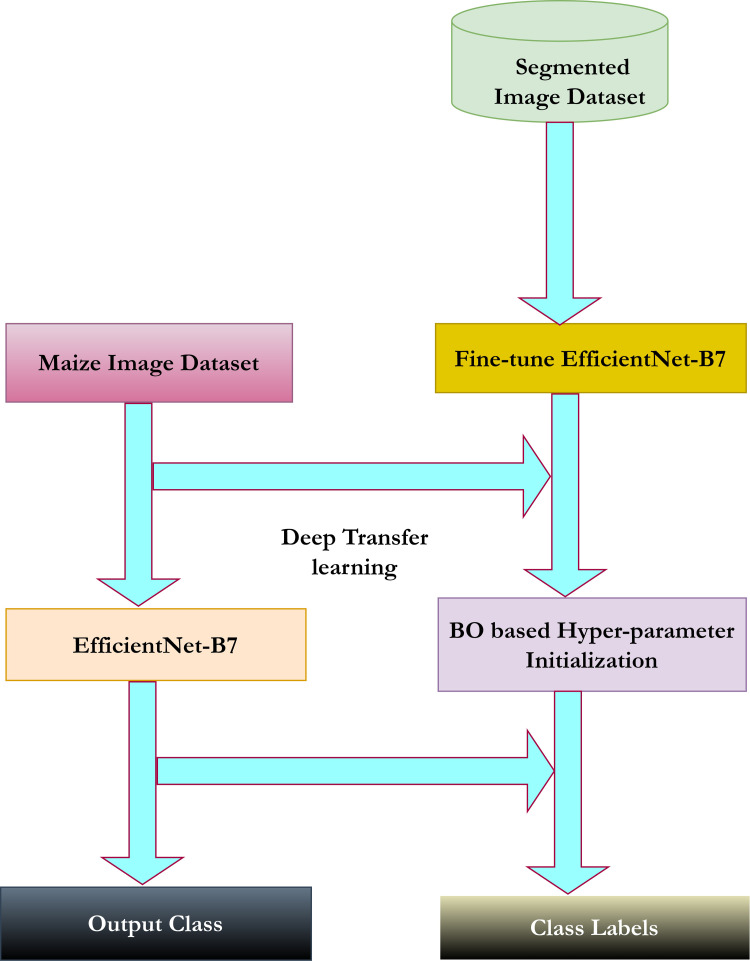
Deep transfer learning with Bayesian hyper-parameter optimization.

EfficientNet-B7 [45] is used for feature extraction in deep learning due to its ability to enhance performance by uniformly scaling across three dimensions, achieving higher accuracy than conventional object recognition models. Despite the typical challenges of high computational time and complex architectures in deep learning models, EfficientNet-B7 is more understandable and underscores the importance of transfer learning. As the number of convolutions increases, so does the network’s depth, leading to more accurate feature extraction. The filters range in size from 16 to 32, 64, and 128, and the ReLU activation function is applied. A global average pooling layer integrates all these layers, followed by a softmax classifier for classification, producing an output of 1000 different object classes. Fine-tuning is crucial and is achieved through deep transfer learning. Fig 4 depicts the deep transfer learning process and the BO technique used for optimizing hyper-parameters, which typically include a static learning rate of 0.0001, momentum ranging from 0.5 to 0.8, and L2 regularization. This optimization process results in a newly trained model specific to maize disease classes.

### 3.6 Image feature selection

The proposed methodology employs Harris Hawks Optimization (HHO) combined with the simulated annealing technique. HHO enhances population diversity in the search space, while simulated annealing enhances HHO’s ability to exploit resources.

#### 3.6.1 Harris Hawks Optimization (HHO).

Harris Hawks Optimization (HHO) is determined by the collaborative hunting behavior of hawks, who work together to confuse their prey. The hawks employ various attacking styles based on their strategy and the prey’s escape patterns. HHO operates in four distinct phases: initialization, exploration, transition, and exploitation.

i) **Initialization Phase**

Initially, the search space and desired function are established, and then all the required attributes are configured to execute the algorithm.

ii) **Exploration Phase**

Initially, the candidate solutions are considered as Harris Hawks. Depending on the prey’s location, the fitness value is updated to find possible solutions. The exploration phase of the Harris Hawks is represented by equation (2) as follows:


$$\begin{array}{c} J(t+1)=\left\{ \;\;\;\begin{array}{c} J_{rand}(t)-r_{1}|J_{rand}(t)-2r_{2}J(t);p\geq 0.5\\ \left(J_{rabbit}(t)-J_{n}(t)-r_{3}\left(lowerbound+r_{4}(upperbound-lowerbound)\right)\right)p<0.5 \end{array}\right. \end{array}$$ (2)

In the second iteration of (t), J(t + 1) the location of the hawks is determined. J_rabbit_ (t) represents the prey’s position and also denotes the randomly selected solution within the population. J(t) is a vector indicating the hawk’s position in the current iteration (t). The variables $r_{1}$, $r_{2}$, $r_{3}$, $r_{4}$ and q represents the scaling factors randomly chosen between 0 and 1, which are updated with each iteration. The upper and lower bounds can be determined in two ways: 1) The answer is determined by selecting a hawk at random from the present population, in addition to other hawks, and using their characteristics to define the solution., or 2) Generating a possible solution based on the hawk’s position. The value of $r_{4}$ is close to 1, while $r_{3}$ is a scaling factor that increases the arbitrary rule. According to this rule, each arbitrary movement length is constrained by the lower bound. This arbitrary scaling factor provides a diverse range of regions inside the feature space. The mean position of the hawks (solutions) is represented by equation (3) as follows:


$$J_{n}(t)=\frac{1}{N}\sum_{i=1}^{N}J_{i}(t)$$ (3)

iii) **Transition Phase:** Based on the prey’s energy (E), HHO posits that the energy fluctuates with each change in the escape pattern due to the Harris hawks’ attack. The prey’s energy is directly linked to its escape pattern. The energy decreases within the range of [-1.1]. If $E_{0}$ is assumed to be the initial energy, then the decrease in energy according to the escape pattern can be expressed by equation (4) as follows:

$$E=2E_{0}\left(1-\frac{t}{T}\right);whereE_{0}\in [-1,1]$$ (4)

In equation (4), T represents the maximum number of iterations, while t denotes the current iteration.

iv) **Exploitation Phase:** This stage is completed using four distinct methods: gentle encirclement, hard encirclement, soft encirclement with gradual hard attacks, and hard encirclement with gradual hard attacks. These procedures have their basis upon two factors: r and |E|. Here, |E| represents the prey’s escaping energy, while r denotes the probability of escaping. If r < 0.5, the prey escapes successfully, and if r ≥ 0.5, the escape attempt is unsuccessful. In the soft besiege approach, when r ≥ 0.5 and |E| ≥ 0.5, it indicates that the prey, although possessing some energy to escape, may lose energy as the hawk approaches for a surprising pounce. The soft besiege can be formulated using equations (5), (6), and (7) as follows:

$$J(t+1)=\Delta J(t)-E\left|{QJ}_{rabbit}-J(t)\right|$$ (5)

$$\Delta J(t)=J_{rabbit}-J(t)$$ (6)

$$Q=2\left(1-r_{5}\right);r_{5}\in [0,1]$$ (7)

ΔJ(t) Denotes the position related to the attack, represented as a vector between the hawk and the prey during the current iteration t. Q defines the energy of the pounce made by the prey, with $r_{5}$ being the random variable. In the case of a hard besiege, when r ≥ 0.5 and |E| < 0.5, it implies that there is a lower chance of escape for the prey. This can be expressed using |E| in equation (8) as follows:


$$J(t+1)=J_{rabbit}(t)-Q\left|\Delta J(t)\right|$$ (8)

When there is a gradual encirclement with a steady decline, where r < 0.5 and |E| ≥ 0.5, it indicates that the prey has sufficient energy to escape. Before making a surprise attack, the hawk patiently waits and advances towards the prey. The hawks approach the prey by determining its position, which can be represented by (9) as follows:


$$M=J_{rabbit}(t)-E\left|{QJ}_{rabbit}-J(t)\right|$$ (9)

The hawk’s decision to dive is directly influenced by its previous dive and its success in catching the prey. Otherwise, the hawks will perform an unpredictable dive, which can be described using the Levy Flight method as shown in equation (10) below:


γ=M+B*LevyFlight(D) (10)

Here, D represents the potential number of solutions; B is a random vector of size 1*D, and Levy Flight(D) refers to the function used to calculate velocity [53]. The Levy flight can be computed using equation (11) as follows:


$$LevyFlight(D)=0.01*\frac{\mu *\lambda }{|\upsilon |\frac{1}{\delta }};\lambda =\left(\frac{H(1+\delta )*\sin\left(\frac{\pi \delta }{2}\right)}{H\left(\frac{1+\delta }{2}\right)*\delta *2\left(\frac{\delta -1}{2}\right)}\right)\frac{1}{\delta }$$ (11)

Here, δ is a constant set to 1.5, and μ and ν are random values between 0 and 1. The position of the Harris hawks is updated with quick, progressive dives, which can be described by equation (12) as follows:


$$\begin{array}{c} J(t+1)=\left\{ \begin{array}{cc} M, & f(M)<f(J(t))\\ \gamma , & f(\gamma )<f(J(t)) \end{array}\right. \end{array}$$ (12)

Equations (9) and (10) update both M and J, representing the new positions in the subsequent iterations.

The final strategy involves an intense encirclement combined with rapid, progressive dives, occurring when r < 0.5 and |E| < 0.5. Once the hawk captures the prey, the rabbit will have no energy left to escape. This hawk attack pattern is described by equation (13) as follows:


$$\begin{array}{c} J(t+1)=\left\{ \begin{array}{cc} M, & f(M)<f(J(t))\\ \gamma , & f(\gamma )<f(J(t)) \end{array}\right. \end{array}$$ (13)

The variable M is initially defined by equation (14) and is further updated according to equation (15) as follows:


$$M=J_{rabbit}(t)-E|QJ_{rabbit}(t)-J_{n}(t)$$ (14)

γ=M+B*LevyFlight(D) (15)

#### 3.6.2 Simulated Annealing (SA).

**Fig 5:**
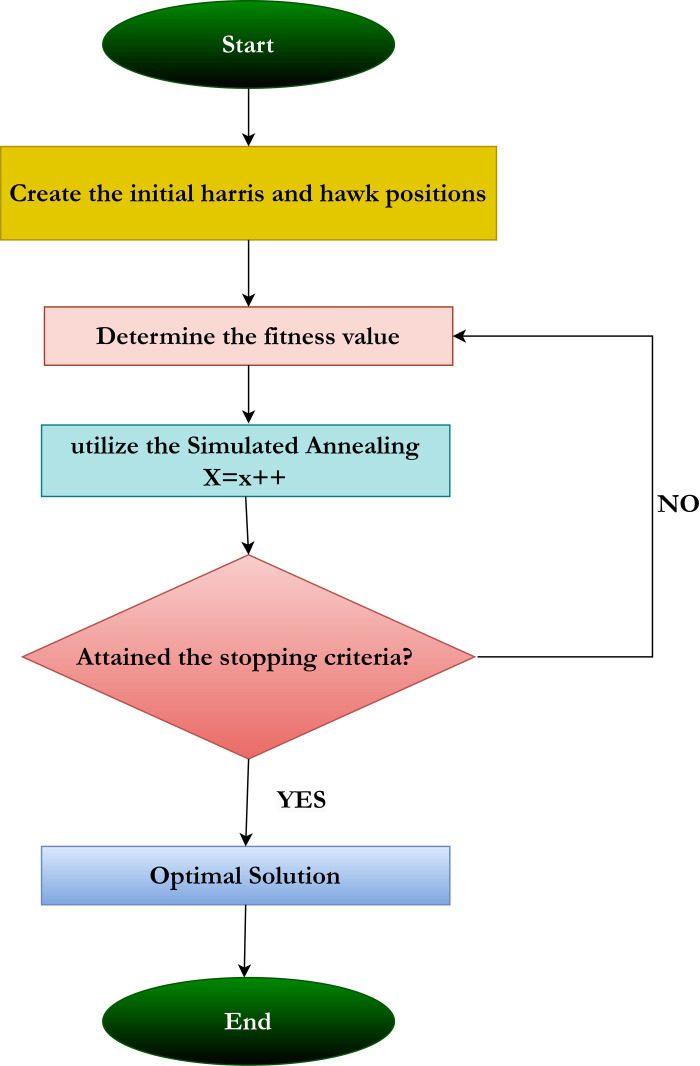
Flow diagram for feature selection with HHOSA.

It utilizes a local search method that yields a single, unique solution, employing a hill-climbing strategy to iteratively find the optimal solution among the existing ones as defined by the objective function [54]. To overcome local optima, the worst solution is considered the best, using the Boltzmann probability function P = e−∅T, where ∅ represents the objective function, indicating the variance between the optimal and least solutions, and T denotes the temperature, which gradually decreases. The algorithm outlines the steps for the proposed hybrid Harris hawk’s optimization algorithm combined with the simulated annealing technique. Fig 5 presents the flowchart for the proposed feature selection method. The working model of the proposed hybrid feature selection technique has been described using Algorithm 1.

**Algorithm 1. Simulated Annealing Hybrid Harris Hawks Optimisation Algorithm.**

**Input:** Size of the Population N and the total Iterations in number T

**Output:** Prey’s position and fitness value

**Step 1:** Establishing the population where Ji; where i = 1,2,3,4…. N

**Step 2:** While (fitness value ≠ stopping criteria), execute

**Step 3:** Calculate the objective function for the hawks

**Step 4:** Set the initial best location of the rabbits as $J_{rabbit}$

**Step 5:** for each hawk (Ji), perform

**Step 6:** Initialize the Prey’s starting energy to $E_{0}$ and the pouncing factor to Q.

**Step 7:** $E_{0}$=2rand ()-1; Q=2(1-rand ())

**Step 8:** The value E is revised using equation (4):- Exploration Phase

**Step 9:** if (|E|≥1) then

**Step 10**: To update the state associated with the vector, apply the equation (2).

**Step 11:** if(|E|<1) then:- Exploration Phase

**Step 12:** if (r ≥ 0.5 and |E|≥0.5) then:- Soft Besiege

**Step 13:** To update the state of the vector, use equation (5)

**Step 14:** if (r ≥ 0.5 and |E|<0.5) then:- Hard Besiege

**Step 15:** To update the state of the vector, use equation (8)

**Step 16:** if (r < 0.5 and|≥0.5) then:- Soft Besiege with progressive dive

**Step 17:** To update the state of the vector, use equation (12)

**Step 18:** else if (r < 0.5 and | E|<0.5) then:- Hard Besiege with progressive dive

**Step 19:** To update the state of the vector, use equation (13)

**Step 20:** Use SA

**Step 21:** T=t++

**Step 22:** Return $J_{rabbit}$

### 3.7 Feature classification using SVM

After the features are classified, the best optimal feature is selected by using the fuzzy SVM classifier. The classifier classifies the maize disease into seven different categories, especially eye spot, downy mildew, goss’s bacterial wilt, Phaeosphaeria leaf spot, gray leaf spot, northern leaf blight, common rust, and healthy image. Since it is a multi-class classification problem, SVM aims to discover the best hyper plane which provides the partition on the features which maximizes the classification origin.

### 3.8 Severity assessment using fuzzy rule-based SVM classifier

Both classification and severity assessment work hand-to-hand which forms the basis of machine learning and data analysis which categorize various lesions based on the fuzzy SVM. To address the innate fuzziness and the suspicion in the real-world image data, fuzzy SVM opts for soft categorization which efficiently overcomes errors and colliding boundaries. The working model of the Fuzzy SVM for both classification and severity assessment is defined in Algorithm 2 as follows:

Based on the works [55–57], the fuzzy rules are defined as projected in Table 5. The fuzzy rules required for the severity assessment involve the use of data obtained from the estimation of lesion density and diseased image of a diseased maize leaf image. The formula to estimate the diseased area and lesion density is given by the equations (16) as follows:

**Table 5:** Fuzzy rule algorithm for severity assessment.

Disease Area	Lesion Density	Disease Accuracy	Severity Index
Low	Low	Low	Negligible
Low	Low	Medium	Mild
Low	Low	High	Severe
Low	Medium	Low	Mild
Low	Medium	Medium	Mild
Low	Medium	High	Severe
Low	High	Low	Severe
Low	High	Medium	Severe
Low	High	High	Severe
Medium	Low	Low	Negligible
Medium	Low	Medium	Mild
Medium	Low	High	Mild
Medium	Medium	Low	Mild
Medium	Medium	Medium	Mild
Medium	Medium	High	Mild
Medium	High	Low	Severe
Medium	High	Medium	Severe
Medium	High	High	Severe
High	Low	Low	Mild
High	Low	Medium	Mild
High	Low	High	Severe
High	Medium	Low	Severe
High	Medium	Medium	Severe
High	Medium	High	Severe
High	High	Low	Severe
High	High	Medium	Severe
High	High	High	Severe


$$LesionDensity(LD)=\frac{\Sigma I_{lesion}}{N_{lesion}}-\frac{\Sigma I_{reference}}{N_{reference}}$$ (16)

From the equation (16) $I_{lesion}$ defines the intensity of pixels within the lesion; $N_{lesion}$ defines the number of pixels within the lesion; $I_{reference}$ defines the pixel intensity inside the reference region$,N_{reference}$ denotes the number of pixels in the reference region. To perform the calculation of the diseased area the region of interest (ROI) is calculated. The formula to compute the diseased area can be defined by the equation (17) as follows:

**Algorithm 2. Severity assessment.**

**Input:** Extracted Features

**Output:** Class (C) and Severity Index (SI)

**Step 1:** Let the classes of each maize disease leaf images are {*C*_1_, *C*_2_, *C*_3_, ..., *C*_*n*_}

**Step 2:** Initialize DA, LD, ACC→low

**Step 3:** Initialize a list *L*_*i*_ according to the total number of features

**Step 4:** For each any image *I*_*i*_; iff (class label==missing)

**Step 5:** then proceed to data pre-processing

**Step 6:** For each class image *C*_1_=rand (0,1)

**Step 7:** Class_state=Low||Medium||High

**Step 8:** iff (*C*_*i*_ < threshold) then

**Step 9:** Class Image is Healthy and SI is set to negligible

**Step 10:** Else SI = FuzzySVM (DA, LD, ACC)

**Step 11:** End iff

**Step 12:** End for

**Step 13:** Then the image is stored in the classified list

**Step 14:** Update *L*_*i*_ + +

**Step 15:** Iff (*C*_*i*_ == *C*_*n*_&&*L*_13_ == *LC*_*n*_)

**Step 16:** SI estimation gets stopped


$${DiseasedArea(DA)=N}_{Diseased}*PixelArea$$ (17)

From the equation (17), $N_{Diseased}$ defines the area of pixels within the diseased region determined by the number of pixels and their pixel area. The overall process of the severity assessment of maize leaf disease can be represented as follows:


$$SI=\frac{1}{2}\{{\left\|W\right\|}^{2}+ACC*\sum \sum C_{i}(LD)*\sum \sum C_{i}(ACC)*SI(DA,LD,ACC)$$ (18)

SI(DA,LD,ACC)=DA*LD*(W*φ(DA*LD)+ACC)≥1 (19)

The severity assessment has been carried out using three levels namely high, medium, and low. These levels depict the severity of the disease affecting the maize plant leaf. When the percentage of severity index ranges between 1–35% then the leaf is negligibly affected and when the range lies between 36–69% then the leaf is mildly affected; when the SI range lies between 70–100% then the leaf gets diseased severely. Table 6 provides the severity levels concerning the percentage of disease affecting each leaf.

**Table 6:** Description of severity level.

Severity Levels	Percentage
Low	1-35%
Medium	36-69%
High	70-100%

## 4. Performance metrics

In order to assess the efficacy of the proposed project, performance metrics such as accuracy, sensitivity, recall, specificity, and F1-measure are used. Accuracy and AUC are the metrics involved in the detection and multi-class classification of maize diseases. The definitions of the performance metrics utilized in our proposed work are as follows:

i) **True Positives (TPs):** The feature is classified as true positives when the classifier successfully labels it.
ii) **True Negatives (TNs):** True negatives are those features that fall under negative classification if the classifier deems them to be negative.
iii) **False Positives (FPs):** When the classifier incorrectly classifies then the feature belongs to True Negatives
iv) **False Negatives (FNs):** False negatives are produced when positive features are classified as negative by the classifier.
v) **Accuracy (ACCY):** It can be defined as the rate of the TPs and TNs to that of all true positives and negatives in sum with the false positives and negatives. Accuracy can be defined by the equation () as follows:

$$ACCY=\frac{TPs+TNs}{TPs+TNs+FPs+FNs}$$ (20)

vi) **Sensitivity (SEY)/Recall:** It can be defined as the rate of the true positives to that of the true positives and false negatives. Sensitivity can be defined by the equation () as follows:

$$SEY=\frac{TPs}{TPs+FNs}$$ (21)

vii) **Specificity (SPY):** Specificity is the ratio of true positives to the sum of true positives and false positives. Specificity may be precisely described using an equation () as follows:

$$SPY=\frac{TPs}{TPs+FPs}$$ (22)

viii) **F1-Measure:** The term refers to the ratio of the product of precision and recall to the sum of precision and recall and further multiplied by 2. The equation that defines F1-Measure can be defined by the equation () as follows:

$$F1-Measure=2*\frac{Precison*Recall}{Precision+Recall}$$ (23)

## 5. Experimentation and analysis

From this equation, the TPs are given by the images which are affected by the maize disease. TNs are the images that are not affected by the maize disease. FPs define the confirmation of the maize leaf disease but are not present. FNs define the confirmation absence of maize leaf disease as present.

### 5.1 implementation and hyper-parameter setting

The proposed methodology has been experimented with in a Tensorflow environment using the python programming language. The experiment was conducted using NVID DGX GPU servers equipped with 512GB of RAM and 8 high-speed Tesla V100 graphics processing units (GPUs), each with a capacity of 32 GB. The CNN model employs the ‘Adam’ optimizer function and the categorical cross-entropy loss function. The learning rate for the Adam optimizer is set at 0.1. Table 7 outlines the specific hyper-parameters that were used in our investigation.

**Table 7:** Hyper-parameters utilized for our proposed work.

Name	Hyper-parameter
Optimization algorithm	Adam
Loss function	Categorical cross-entropy
Leaning rate	0.001
Momentum	0.9
Weights decades	0.004
Epochs	1000
Batch size	32

The images that are acquired and resized to 227*227-pixel size for an optimized performance. For each batch of training of image, a tensor gets created in Keras using the image generator package. This package is utilized to eliminate the problem of overfitting. The major advantage of our proposed technique is that it reduces the training time or learning time since the images are augmented. Therefore, no extra storage of these pre-processed augmented images.

### 5.2 Confusion matrix

Within the context of the testing dataset, a confusion matrix is used to define the performance features of the proposed model. Rows define the class and the columns define prediction values. The diagonals represent the accurate predictions while the off-diagonals represent the false predictions.

## 6. Results and discussion

### 6.1 The proposed methodology’s predictive performance

In accordance with the percentage 80:20, a dataset that will be utilized for the approach that we have presented has been divided into three sections. From the 80% of data, the dataset has been split into 70% for training and 10% for validation and 20% for testing. The proposed deep-saliency-based segmentation along with EfficientNetB7 are trained and validated for 1000 epochs with 32 batch sizes per epoch. The proposed methodology has been evaluated based on training and validation accuracy, and losses. To evaluate the performance of the proposed model the categorical cross-entropy loss function and model accuracy were utilized on the test data. Therefore, the categorical cross-entropy loss function can be defined using the equation (20) as follows:


$$Loss=-\frac{1}{n}\sum_{i=1}^{n}y_{i}\log{\hat{y}}_{i}$$ (24)

From the equation (24)$y_{i}$ provides the targeted class output; ${\hat{y}}_{i}$ defines the estimated output vector of the ith sample within a mini-batch of n samples. The output of the prediction can be correct or incorrect.

**Fig 6:**
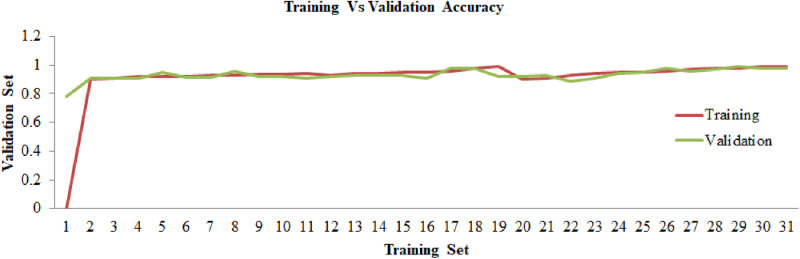
Training vs validation accuracy.

**Fig 7:**
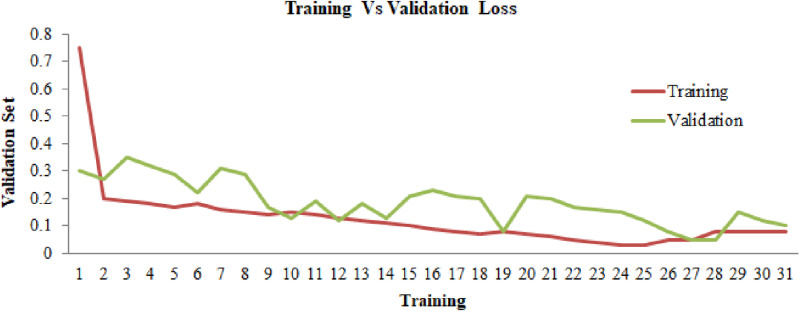
Training vs validation loss.

Figs 6 and 7 provides the training and validation accuracy and losses for the proposed model. The components of accuracy projected a considerable rise when the training epoch increases whereas the component of loss projected a considerable tumble as the training epoch increases. The EfficientNetB7 model obtained 99.47% overall classification accuracy and a loss of 0.0053. The proposed approach exhibited exceptional performance on the testing dataset. The performance findings demonstrate that the suggested model is very well-suited for accurately identifying illnesses in maize leaves.

### 6.2 Analysis based on confusion matrix

**Fig 8:**
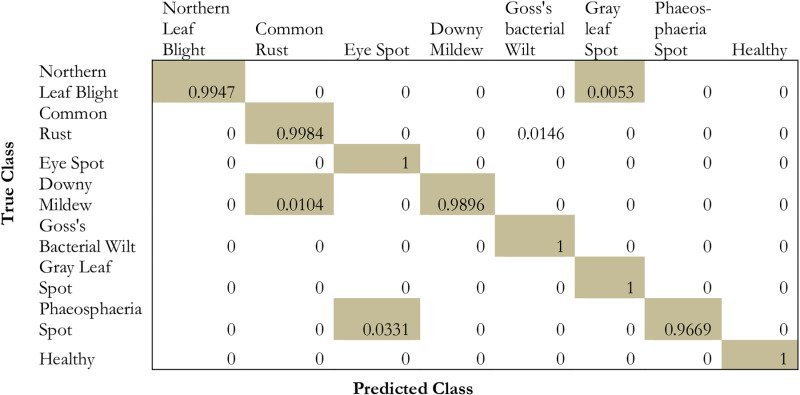
Confusion matrix for the proposed methodology.

The evaluation is done on the model by using the confusion matrix on the testing dataset which is depicted in Fig 8 and Table 8.

**Table 8:** Prediction accuracy and misclassification rate.

Classes	Accuracy	Misclassification Rate
Northern Leaf Blight	0.9947	0.0053
Common Rust	0.9987	0.0146
Downy Mildew	0.9896	0.0104
Phaeosphaeria leaf spot	0.9669	0.0331

From the table and the Fig 8, it is evident that the model projects a high prediction performance on eye spot, goss’s bacterial wilt, gray leaf spot, and healthy images. However, the model projected medium performance in the case of common rust, Phaeosphaeria leaf spots, downy mildew, and northern leaf blight images. Similarly, the class GLS performance very little in the recognition of maize leaf disease.

### 6.3 Comparative analysis based on various deep learning models

**Fig 9:**
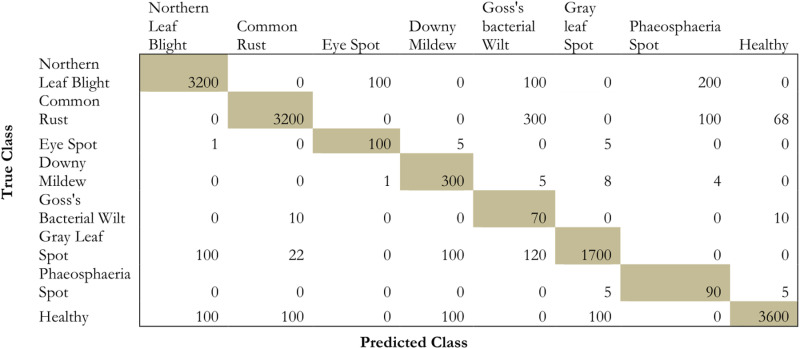
Provides the prediction performance in terms of a confusion matrix based on ResNet5.

**Fig 10:**
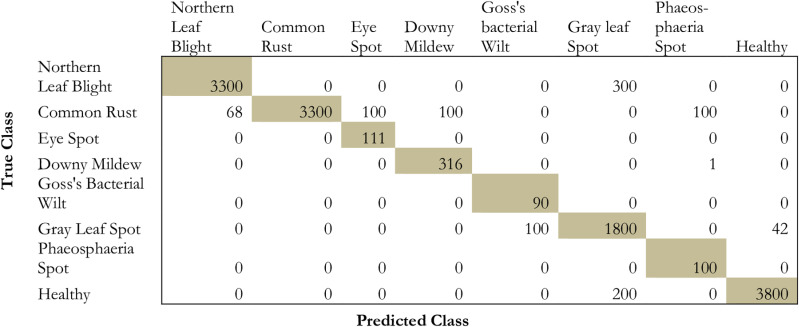
Provides the prediction performance in terms of confusion matrix based on ResNet152.

**Fig 11:**
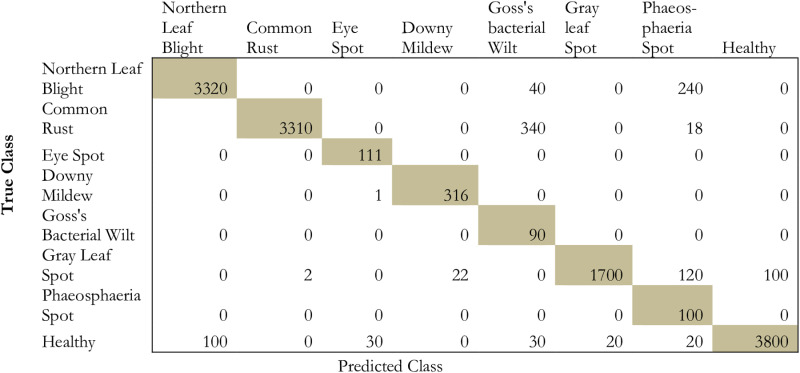
Provides the prediction performance in terms of the confusion matrix based on InceptionV3.

**Fig 12:**
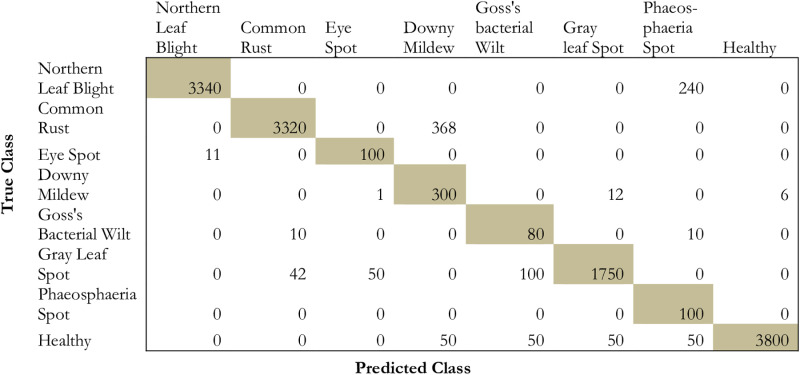
Provides the prediction performance in terms of the confusion matrix based on DenseNet152.

**Fig 13:**
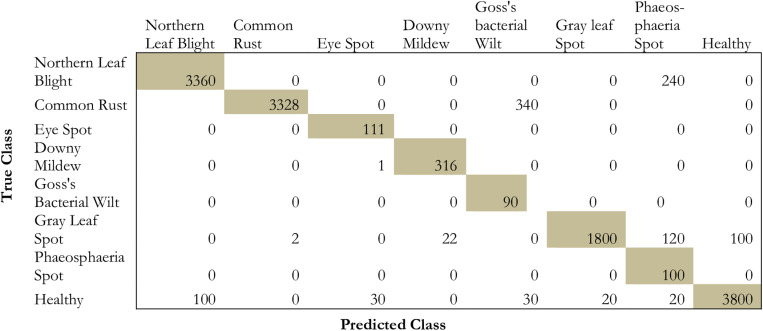
Provides the prediction performance in terms of the confusion matrix for the proposed model based on EfficientNet-B7.

The proposed model has been compared to conventional deep transfer learning models such as ResNet152, DenseNet152, InceptionV3, and ResNet50. Fig 9–13 projects the comparative analysis of various classes of maize disease concerning different transfer learning models.

From the comparative analysis in terms of the confusion matrix, it is obvious that the proposed model exhibits a significant performance in identifying the false positives, false negatives, and true negatives. From Figs 9–13 the proposed model shows high performance followed by densenet152, inceptionv3, resnet152, and resnet50. Due to the presence of hyper-parameter optimization and simulated annealing technique, the convergence for repeated oscillation towards true negatives and false positives is considerably reduced.

### 6.4 Comparative analysis in terms of class-wise prediction performance of the model proposed

From Table 9 the proposed model achieves high performance > 98% on all classes to classification accuracy and specificity. However, classes such as eyespot, goss’s bacterial wilt exhibit less specificity due to the presence of false negatives and northern leaf blight exhibits less performance in terms of sensitivity due to the presence of true negatives. However, this comparative analysis in terms of class-wise results is inadequate since it suffers from class imbalance problems. Therefore, the F1-Score is used as the metric to assess the model’s performance where goss’s bacterial wilt, gray leaf spot, Phaeosphaeria leaf spot, and healthy leaf images exhibit a significant performance >99%. However northern leaf blight and eye spot show lowered performance. Therefore, it is evident that the proposed model is sufficient to identify maize diseases such as common rust, bacterial wilt, gray leaf spot, Phaeosphaeria leaf spot, and healthy leaves. Therefore, the proposed deep transfer learning model achieved an F1-Score of 98.55% implying that suitability for recognizing maize diseases. The figure demonstrates that the suggested model attained an F1-score of 98.55% also a classification accuracy of 99.47%, having a loss of 0.0145. These findings indicate that the classification accuracy improves as the number of epochs grows during testing. Thus, the proposed model exhibits high prediction accuracy.

**Table 9:** Comparative analysis of class-wise prediction performance.

Class	TP	FP	FN	TN	Accuracy (%)	Specificity (%)	Sensitivity (%)	Precision (%)	F1-Score (%)
Northern Leaf Blight	3360	0	0	240	98.83	97.52	98.15	100	95.88
Common Rust	3328	0	0	340	98.56	97.88	98.33	100	100
Eye Spot	100	0	5	6	97.55	100	100	98.27	96.52
Downy Mildew	300	0	1	16	99	100	100	99.63	98.12
Goss’s Bacterial Wilt	80	2	5	3	97.83	100	100	99.45	99.88
Gray Leaf Spot	1800	2	40	200	97.66	98.55	99.6	100	99.52
Phaeosphaeria leaf spot	90	0	0	10	100	100	100	99.48	99.43
Healthy	3800		0	200	99.99	98.83	99.94	100	99.1
Average					98.67	99.09	99.12	99.6	98.55

### 6.5 Comparative analysis in terms of performance metrics based on transfer learning models

From the chosen deep transfer learning models ResNet50, ResNet152, InceptionV3, and DenseNet152, VGG19 an analysis has been performed to evaluate the effectiveness of the proposed model. ImageNet data [58] has been utilized for training and validation. Learning weights are chosen and applied for the classification of the maize diseases. Table 10 projects the performance of the existing deep transfer learning models to that of the proposed model. It is observed all the deep transfer learning models are good in the classification of the test data with more than 90% accuracy except ResNet50 and VGG19.

**Table 10:** Comparative analysis of transfer learning models.

Model	Testing Accuracy (%)	Testing Loss	Average Precision (%)	Average Sensitivity (%)	Average F1-Score
ResNet50	99.05	0.0989	97.35	97.29	97.17
ResNet52	99.16	0.1613	98.72	97.54	96.22
InceptionV3	98.12	0.2102	93.66	95.44	95.20
DenseNet152	98.50	0.2227	94.66	93.59	93.39
VGG19	99.28	0.0554	96.37	95.67	95.10
EfficientNetB0	93.39	0.33331	88.12	90.33	88.04
EfficientNetB7	97.93	0.1369	92.55	91.89	93.07
Deep Saliency+EfficientNetB7	99.47	0.0153	98.63	98.55	98.55

From the Table 10 it is observed that VGG19 achieved the highest accuracy of 99.28% followed by ResNet152 of 99.16% occupying the second highest performance. EfficientNetB0 occupies the last with a least accuracy of 93.39% followed by EfficientNetB7 of 97.93%. Similarly, according to F1-Score ResNet50 shows the highest performance followed by ResNet152 exhibiting the second highest performance. From the table, it is obvious that efficientNetB0 exhibits a lower performance in terms of F1-Score. Thus, it is evident that the proposed model shows 99.47% accuracy with an improvement in prediction performance and 98.63% precision when compared to that of the other deep transfer learning models for the classification of maize diseases.

**Fig 14:**
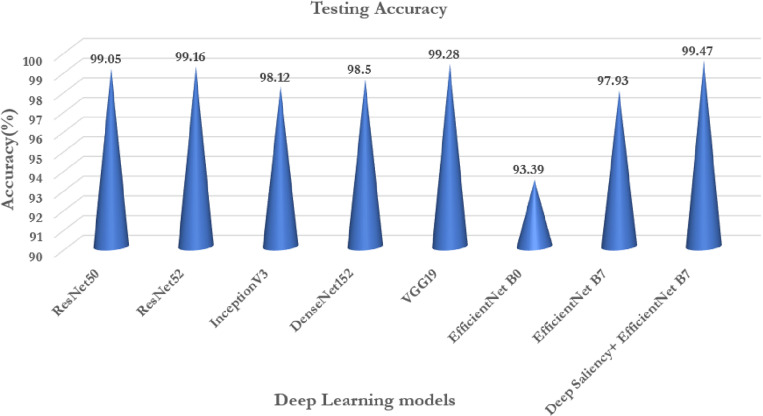
Comparative analysis of performance metrics of the proposed and the deep transfer learning models in terms of classification accuracy.

**Fig 15:**
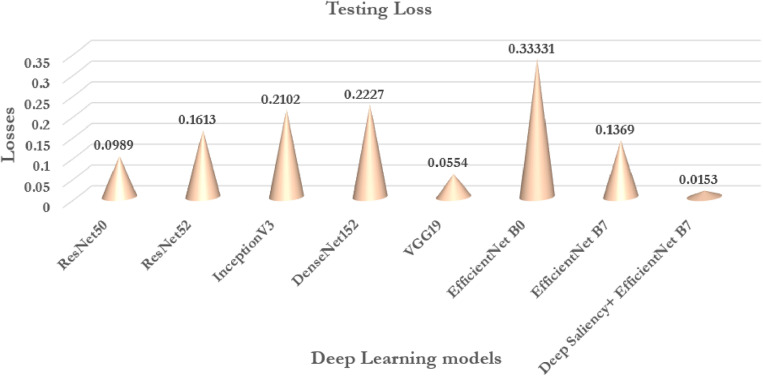
Comparative analysis of performance metrics between the proposed models and deep transfer learning models in terms of testing loss.

**Fig 16:**
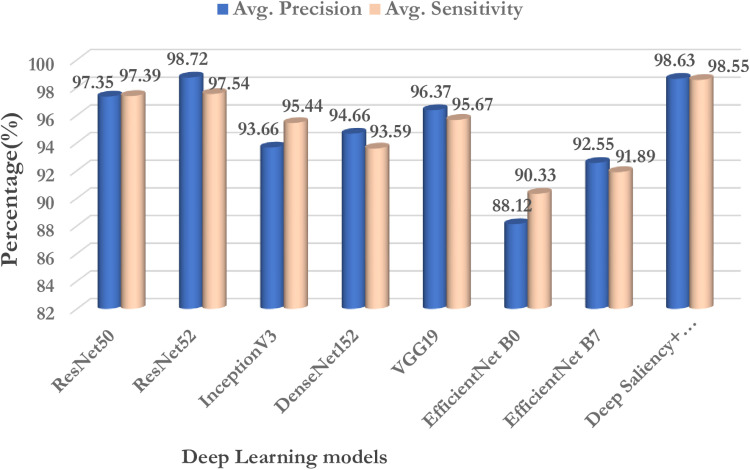
Comparative analysis of performance metrics of the proposed and the deep transfer learning models in terms of average precision and sensitivity.

**Fig 17:**
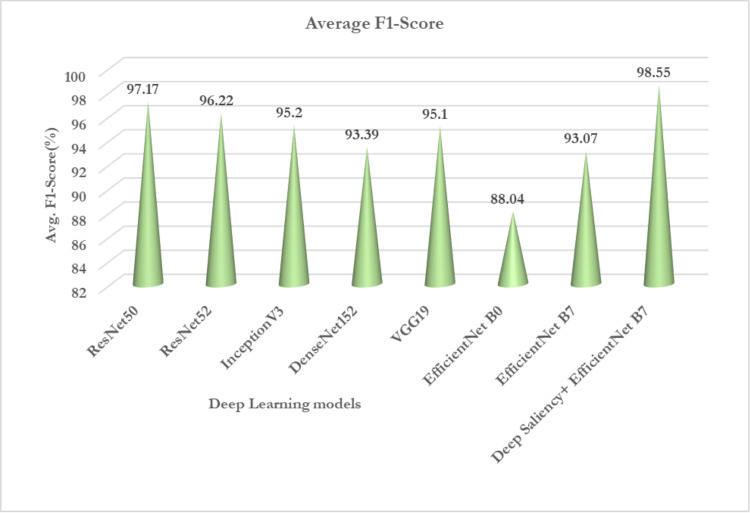
Comparative analysis of performance metrics of the proposed models and deep transfer learning models in terms of average F1-score.

Figs 14–17 Comparative analysis of performance metrics of the proposed and the deep transfer learning models in terms of classification accuracy, loss, specificity, sensitivity, and F1 scores.

#### 6.5.1 Classification accuracy and loss.

From Graph 14), 15) it is evident that the proposed deep saliency-based segmentation and classification model achieved higher classification accuracy than the best promising deep transfer learning model, i.e., the VGG19 model. The testing loss in the case of our proposed work exhibits 0.0153 which is the least in comparison to that of the pre-defined models. Thus, the evaluation proves that the proposed deep saliency-based segmentation and classification model is better than the state-of-the-art pre-trained deep transfer learning models of classification accuracy and loss.

#### 6.5.2 Average F1-scores.

Even using data augmentation techniques, the dataset still exhibits class imbalance, which involves a comparison study of all models with respect to performance indicators such as F1-score, recall, and precision. Figs 16 and 17 from the analysis carried out proves that the proposed deep saliency segmentation and classification model achieves a higher F1-score of 98.55% than that of the other pre-trained models. Thus, deep saliency maps reduced half of the feature selection in images reducing the computation overhead incurred by the deep transfer learning models. Since a separate hybrid Harris Hawks’ optimization with simulated annealing technique is employed the convergence rate is very low which makes it less prone to error during the classification of maize diseases.

### 6.6 Comparative analysis with the existing systems

The proposed model has been evaluated in terms of classes, dataset, Models, and accuracy which are compared to that of the existing systems Haque et.al., [26], Amin et.al., [25], Thakur et.al.,[24], Singh et.al., [33], Masood et.al., [30]. Table 11 provides a comparative analysis of the performance metrics to that of the existing systems.

**Table 11:** Comparative analysis of prediction performance to that of existing systems.

Ref	Classes	Dataset	Models	Results
[24]	4 Classes	PlantVillage	VGG-ICNN	Accuracy 99.16%
[25]	4 Classes	PlantVillage	EfficientNetB0 + DenseNet121	Accuracy 98.56%
[26]	4 Classes	PlantVillage	CNN	Accuracy 99.1%
[30]	4 Classes	PlantVillage	Faster RCNN	Accuracy 97.89%
Proposed model	8 Classes	PlantVillage	Deep Saliency Map+EfficientNetB7	Accuracy 99.47%

From the Table 11, it can be seen that the proposed model has chosen a greater number of diseases when compared to that of the existing systems which involves optimization algorithms exhibiting an accuracy of 99.47% which is the best in recognizing the unknown or known classes of maize diseases.

## 7. Ablation analysis of the proposed work

The ablation analysis has been conducted for the proposed work which has been aimed to assess 1) The impact of data augmentation in the performance of classification; 2) The impact of amount of unlabeled data volume.

### 7.1 Data augmentation analysis

Experiments are conducted without data augmentation, which highlighted its significant impact on model performance. Single augmentation methods were insufficient, though horizontal flipping alone performed best, enabling the extraction of critical features. The absence of any one of the four augmentation operations resulted in reduced performance, whereas utilizing a combined data augmentation approach incorporating all four operations significantly enhanced the effectiveness of the proposed solution. Table 12 demonstrates that data augmentation had a substantial effect on model performance.

**Table 12:** Comparative analysis of accuracy for different image augmentation techniques.

Rotation (90^0^)	Width shift (0.2)	Hight shift	Horizontal flipping	Accuracy
				88.26
✔				90.62
	✔			91.37
		✔		90.74
			✔	92.41
✔	✔	✔		94.11
✔		✔	✔	95.06
✔	✔		✔	95.91
	✔	✔	✔	97.81
✔	✔	✔	✔	99.47

### 7.2 Influence of unlabeled data volume

The Proposed model is trained on PlantVillage dataset and fine-tuned to evaluate the impact of the quantity of unlabeled data. As illustrated in Table 13, the proposed model trained exclusively on the PlantVillage dataset exhibited a better feature learning, leading to superior classification and segmentation performance when compared to the other existing models trained on datasets of different sizes. This indicates that the proposed model is able to perform well even on the dataset with the limited size compared to the other existing works.

**Table 13:** Comparison of performance of Existing works and Proposed solution with different data volumes.

Reference	Dataset	Image	Accuracy
[24]	PlantVillage	54305	99.16%
	Embrapa	46376	93.66%
	Apple	3642	94.24%
	Maize	400	91.36%
	Rice	500	96.67%
[25]	PlantVillage	217000	98.56%
[26]	PlantVillage	3852	99.1%
[30]	PlantVillage	2112	97.89%
Proposed model	PlantVillage	16118	99.47%

## 8. Conclusion and future work

Maize crop leaf diseases significantly impact both the quality and quantity of maize production, ultimately affecting the market. This research utilizes a deep saliency map segmentation-based CNN for the detection, multi-class classification, and severity assessment of maize crop leaf diseases. By incorporating segmentation techniques, our model accurately identifies and delineates disease-affected regions within maize crop leaf images, enabling precise quantification of disease severity levels and facilitating more informed agricultural management decisions. Leveraging deep CNNs ensures robust disease prediction and multi-class classification capabilities while maintaining computational efficiency. The proposed technique has been shown useful in properly detecting and analysing the severity of leaf diseases in maize crops through experimental research. The proposed work contributes to advancing automated disease diagnosis in agriculture, thereby supporting efforts for sustainable crop yield improvement and food security. The proposed model was tested on seven different classes: northern leaf blight, common rust, eye spot, downy mildew, Goss’s bacterial wilt, gray leaf spot, Phaeosphaeria leaf spot, and healthy images. It achieved higher classification accuracy than the best-performing deep transfer learning models. The testing loss of our proposed model is 0.0153, which is the lowest compared to pre-trained models. Hence, the research demonstrates that our advanced saliency-based segmentation and classification model surpasses the most advanced pre-trained deep transfer learning models in terms of accuracy and loss in classification. Additionally, the proposed method recognizes a greater number of diseases compared to existing systems, achieving an accuracy of 99.47% in identifying maize leaf diseases.

Future research will focus on integrating more effective methods to identify illness in maize leaves into real-time devices, aiming to reduce costs by minimizing the use of pesticides, chemicals, and insecticides. Additionally, this methodology will be adapted to categorize multiple types of infections in various plants, applicable to both the same and different crops. Additionally, the loss incurred during image vision and the problem of distinguishability has to be addressed. Classification accuracy needs to be improved by cascading various deep learning approaches with advanced fusion aware techniques.
